# Can Replacing CA125 with HE4 in Risk of Malignancy Indices 1–4 Improve Diagnostic Performance in the Presurgical Assessment of Adnexal Tumors?

**DOI:** 10.1155/2017/6712376

**Published:** 2017-11-07

**Authors:** Nabil Abdalla, Robert Piórkowski, Paweł Stanirowski, Krzysztof Cendrowski, Włodzimierz Sawicki

**Affiliations:** Department of Obstetrics, Gynecology and Oncology, Second Faculty of Medicine, Medical University of Warsaw, Kondratowicza 8, 03-242 Warsaw, Poland

## Abstract

**Aims:**

To assess whether replacing CA125 with HE4 in the classical formulas of risk of malignancy indices (RMIs) can improve diagnostic performance.

**Methods:**

For each of 312 patients with an adnexal mass, classical RMIs 1–4 were computed based on ultrasound score, menopausal status, and serum CA125 levels. Additionally, modified RMIs (mRMIs) 1–4 were recalculated by replacing CA125 with HE4.

**Results:**

Malignant pathology was diagnosed in 52 patients (16.67%). There was no significant difference in diagnostic performance (area under the receiver operating characteristic curve [AUC]) between each classical RMI and its corresponding mRMI. In the entire sample, the AUC was 0.899, 0.900, 0.895, and 0.908 for classical RMIs 1–4 compared to 0.903, 0.929, 0.930, and 0.931 for mRMIs 1–4. In premenopausal patients, the AUC was 0.818, 0.798, 0.795, and 0.802 for classical RMIs 1–4 compared to 0.839, 0.875, 0.876, and 0.856 for mRMIs 1–4. In postmenopausal patients, the AUC was 0.906, 0.895, 0.896, and 0.906 for classical RMIs 1–4 compared to 0.907, 0.923, 0.924, and 0.930 for mRMI 1–4.

**Conclusions:**

Use of HE4 instead of CA125 did not significantly improve diagnostic performance of RMIs 1–4 in patients with an adnexal mass.

## 1. Introduction

Risk of malignancy indices (RMIs) are multimodal scoring systems used for the presurgical differentiation of adnexal tumors. RMI 1, originally proposed by Jacobs et al. in 1990, is calculated based on ultrasound findings, serum levels of tumor marker cancer antigen 125 (CA125), and menopausal status [1]. In 1996, Tingulstad et al. introduced RMI 2, which modified RMI 1 by replacing the values of the parameters; in 1999, the same authors proposed RMI 3, which further modified the values of the RMI 1 parameters [2, 3]. Finally, in 2009, Yamamoto et al. proposed RMI 4 by including an additional ultrasound parameter in the RMI 1 formula [4].

Currently, many national guidelines for the management of malignancies emphasize the role of RMIs in the preoperative assessment of adnexal tumors [5, 6]. However, a recent meta-analysis by Meys et al. [7] found that subjective assessment is superior to these scoring systems. Specifically, the 47 articles analyzed by Meys et al. described the outcomes of 19,674 adnexal masses assessed via subjective assessment, simple rules, logistic regression models (LR2), and RMIs 1–3. The authors concluded that simple rules with subjective assessment by experienced ultrasound examiners (for inconclusive masses) yielded the best results. When an expert is not available, LR2 can be applied instead of subjective assessment [7]. These findings justify the need for the development of tools based only on ultrasound assessment, eliminating the need for blood taking for tumor marker measurement. Until such tools become available for clinical use, further improvement of current RMIs is desirable.

In 1991, Kirchhoff et al. published the first report describing the presence of human epididymis protein (HE4) in the distal part of the epididymis [8]. In 2003, Hellström et al. were the first to highlight the potential role of HE4 as a serum marker of ovarian cancer [9]. Then, in 2008, Moore et al. concluded that, as a single marker, HE4 had the highest sensitivity for detecting ovarian cancer, especially stage I, among patients with adnexal masses [10]. Similarly, in 2011, Escudero et al. showed that HE4 had higher specificity than CA125 in patients with benign gynecologic diseases, with abnormal concentrations of HE4 and CA125 noted in 1.3% and 33.2% of patients, respectively, and a significantly higher area under the receiver operating characteristic (AUC-ROC) curve for HE4 than for CA125 when differentiating benign from malignant diseases [11]. These findings regarding the high specificity of HE4 in patients with adnexal masses may justify the use of HE4 instead of CA125 in the formulas of RMIs for the presurgical assessment of adnexal masses.

With the above in mind, the aim of the present study was to perform a comparative evaluation of the diagnostic performance of classical RMIs 1–4 against modified RMIs (mRMIs) 1–4 (where CA125 is replaced with HE4) in the preoperative differentiation of malignant from nonmalignant adnexal tumors in premenopausal and postmenopausal patients.

## 2. Materials and Methods

This prospective study included 312 patients admitted to our clinic between October 2012 and May 2015 and scheduled to undergo surgery for adnexal tumors. The inclusion criteria were age ≥ 18 years, ultrasound assessment of adnexal mass and measurement of tumor markers CA125 and HE4 within 5 days before surgical intervention, and informed consent. The exclusion criteria were renal disease, history of malignancy, chemotherapy, radiotherapy, presence of fibroids > 5 cm, and lack of histological assessment of the mass.

Each patient underwent transvaginal ultrasound. Transabdominal ultrasound was performed in patients who were virgins at the time of treatment, when the mass could not be visualized entirely by transvaginal ultrasound, and to detect metastases in the abdominal organs when there was suspicion of malignancy. Examination was performed using the ultrasound apparatus Philips iU22. The following ultrasound findings were considered in the examination: multilocular cyst, solid areas, bilateral lesions, ascites, and metastases [1–4]. The definitions of multilocular cyst, solid areas, and ascites were consistent with the terms and definitions established by the International Ovarian Tumors Analysis (IOTA) group [12]. Distant metastases were defined as focal lesions in renal, splenic, and hepatic parenchyma or as omental cake. If bilateral lesions were noted, only data regarding the lesion with a more complex structure were included in the statistical analysis.

One point was assigned for each ultrasound finding and the sum was used to determine the value for the RMI ultrasound score (*U*). The maximum diameter of the lesion (*S*) was considered as an additional ultrasound parameter in RMI 4 [4]. Menopause was defined as the absence of menstruation for at least 1 year. Serum CA125 and HE4 levels were measured via electrochemiluminescence immunoassay performed using a Cobas 8000 e602 apparatus. The formulas of the classical RMIs 1–3 used the ultrasound score (*U*), menopausal status (*M*), and serum levels of CA125 (RMIs 1–3 = *U* × *M* × CA125), while RMI 4 also included the maximum diameter of the lesion (*S*) (RMI 4 = *U* × *M* × CA125 × *S*) [1–4].

For RMI 1, a *U*-value of 0, 1, and 3 was assigned when the total ultrasound scores were 0, 1, and ≥2 points, respectively. For RMIs 2 and 4, a *U*-value of 1 and 4 was assigned when the total ultrasound scores were ≤1 and ≥2 points, respectively. For RMI 3, a *U*-value of 1 and 3 was assigned when the total ultrasound scores were ≤1 and ≥2 points, respectively. Menopausal (*M*) status had a value of 1 in premenopausal patients and a value of 3 or 4 in postmenopausal patients depending on whether *M* was included in RMIs 1 and 3 or RMIs 2 and 4, respectively. The serum levels of CA125 were used directly in the formulas of classical RMIs 1–4. *S*-value was 1 and 2 for maximal lesion diameters of <70 mm and ≥70 mm, respectively [1–4]. The corresponding modified RMIs 1–4 (mRMI 1–4) were calculated using the same formulas after replacing the serum levels of CA125 by those of HE4 as follows: mRMIs 1–3 = *U* × *M* × HE4 and mRMI 4 = *U* × *M* × HE4 × *S*.

The final diagnosis of adnexal masses was based on histopathological examination of the excised masses. The staging of malignant masses was based on the guidelines of Fédération Internationale de Gynécologie et d'Obstétrique (FIGO). In the statistical analysis, borderline tumors were considered as malignant. All data of tumor markers, menopausal status, and ultrasound features were collected prospectively. Both classical and modified forms were collected and documented in a prospective manner. The final decision about the method of management was taken by at least two gynecologists depending on classical RMIs, serum CA125 levels, and subjective assessment of adnexal tumors. The operators were unaware of the results of mRMIs. At the end of the study, the final analysis was performed. A cut-off of 200 and 450 was set for RMIs 1–3 and RMI 4, respectively, as suggested by the original proponents of each index [1–4]. The sensitivity, specificity, positive predictive value (PPV), negative predictive value (NPV), diagnostic accuracy, positive likelihood ratio (LR+), negative likelihood ratio (LR−), and diagnostic odds ratio (DOR) of the classical and mRMIs were calculated. Sensitivity defines the proportion of truly positive subjects with the disease in a group of all subjects with the disease. Specificity is defined as the proportion of subjects without the disease with negative test results within all subjects without the disease. PPV is defined as the proportion of patients with positive test results in all subjects with positive results. NPV represents the proportion of subjects without the disease with a negative test result in all subjects with negative test results. LR+ can be calculated according to the following formula: LR+ = sensitivity/(1 − specificity). LR− is calculated according to the following formula: LR− = (1 − sensitivity)/specificity. Diagnostic accuracy is expressed as a proportion of correctly classified subjects among all subjects. DOR of a test is the ratio of the odds of positivity in subjects with disease relative to the odds in subjects without disease [13]. Measures of diagnostic accuracy were performed for the differentiation of malignant from nonmalignant adnexal masses in the whole sample (premenopausal and postmenopausal patients). These measures were also used for the differentiation of malignant stage I (FIGO) adnexal tumors from nonmalignant adnexal masses. The Mann–Whitney* U* test was used to assess the differences in the distribution of CA125 and HE4 levels according to the malignancy status of the adnexal mass. The chi-square test was used to assess the differences in the distribution of age, menopausal status, and ultrasound score according to malignancy status. The area under the ROC curve (AUC) was constructed for both classical and mRMI 1–4. The Hanley and McNeil test was used to assess the difference between the AUCs of classical and mRMIs. A *p* value < 0.05 was considered to indicate statistical significance. Diagnostic measures for mRMI were calculated depending on optimal cut-off levels gained from AUC. The study protocol was approved by the local Ethical Committee number KB/192/2012.

## 3. Results

A total of 312 patients were included in the study. Patient age ranged from 18 to 85 years with a mean of 48.5 years (standard deviation, 16.8 years). Malignant pathology was diagnosed in 52 patients (16.67%). A total of 117 (37.50%) patients were postmenopausal at the time of assessment. The distribution of the nonmalignant and malignant pathologies is displayed in Tables 1 and 2, respectively, whereas the distribution of FIGO stages of malignant adnexal pathologies is shown in Table 3.

The differences among nonmalignant and malignant adnexal masses in terms of age, menopausal status, ultrasound score, and tumor diameter are shown in Table 4. The descriptive statistics of the distribution of biomarker serum levels (CA125 and HE4) are shown in Table 5.

The diagnostic performance of classical RMIs 1–4 is shown in Table 6 for the entire study sample and for groups defined in terms of menopausal status. The diagnostic performance of these indices for the differentiation of malignant stage I (FIGO) from nonmalignant adnexal tumors is shown in Table 7.

**Figure 1:**
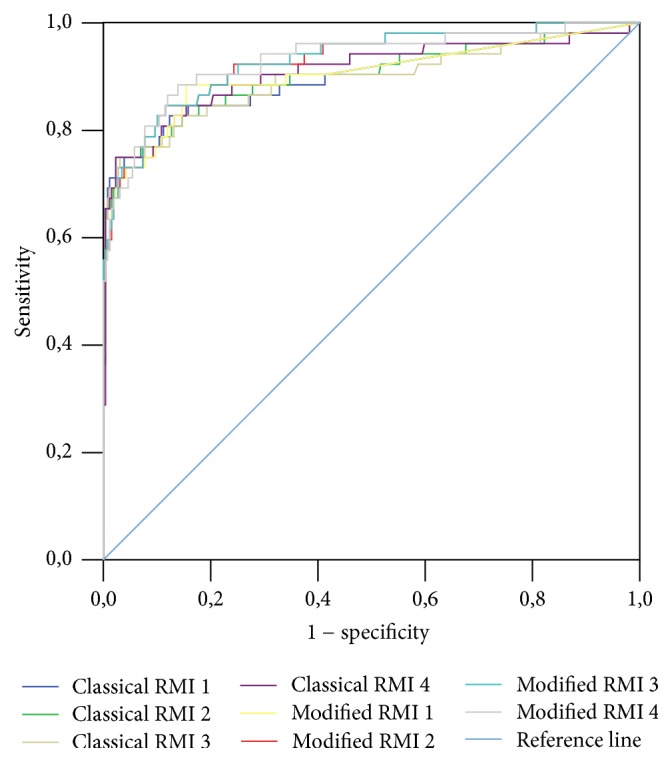
Diagnostic accuracy of classical and modified risk of malignancy indices (RMIs) 1–4 for the entire study sample of patients with adnexal mass. Modified RMIs were obtained by replacing cancer antigen 125 with human epididymis secretory protein 4 in the classical RMIs. Diagnostic accuracy was based on receiver operating characteristic analysis. Diagonal segments are produced by ties.

**Figure 2:**
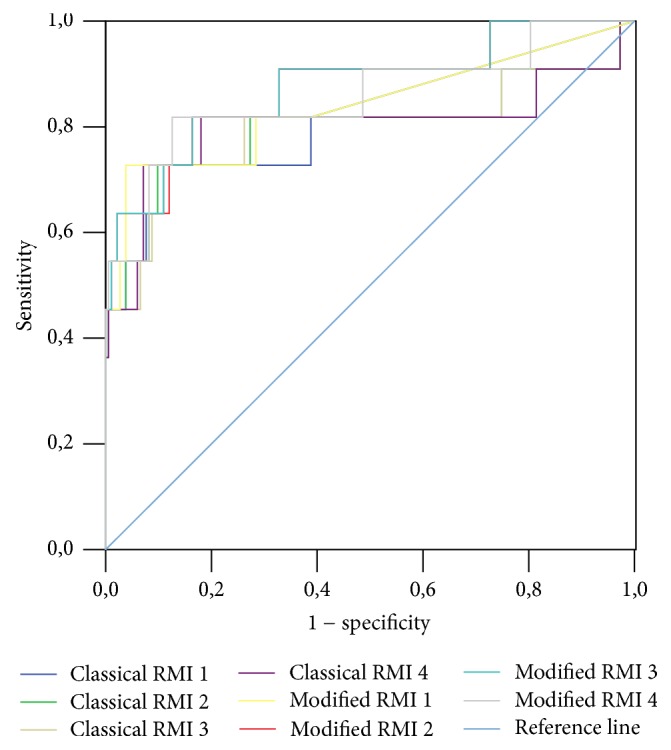
Diagnostic accuracy of classical and modified risk of malignancy indices (RMIs) 1–4 for premenopausal patients with adnexal mass. Modified RMIs were obtained by replacing cancer antigen 125 with human epididymis secretory protein 4 in the classical RMIs. Diagnostic accuracy was based on receiver operating characteristic analysis. Diagonal segments are produced by ties.

**Figure 3:**
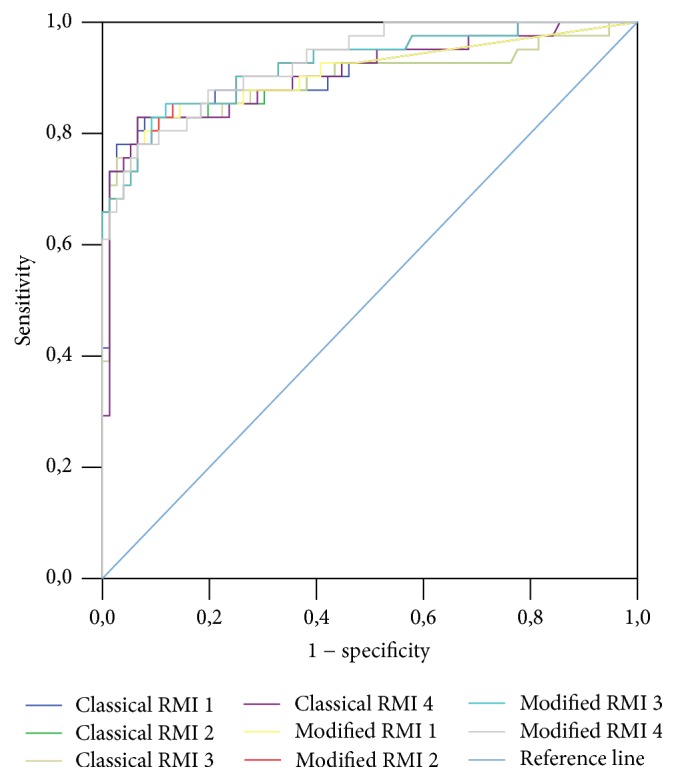
Diagnostic accuracy of classical and modified risk of malignancy indices (RMIs) 1–4 for postmenopausal patients with adnexal mass. Modified RMIs were obtained by replacing cancer antigen 125 with human epididymis secretory protein 4 in the classical RMIs. Diagnostic accuracy was based on receiver operating characteristic analysis. Diagonal segments are produced by ties.

**Figure 4:**
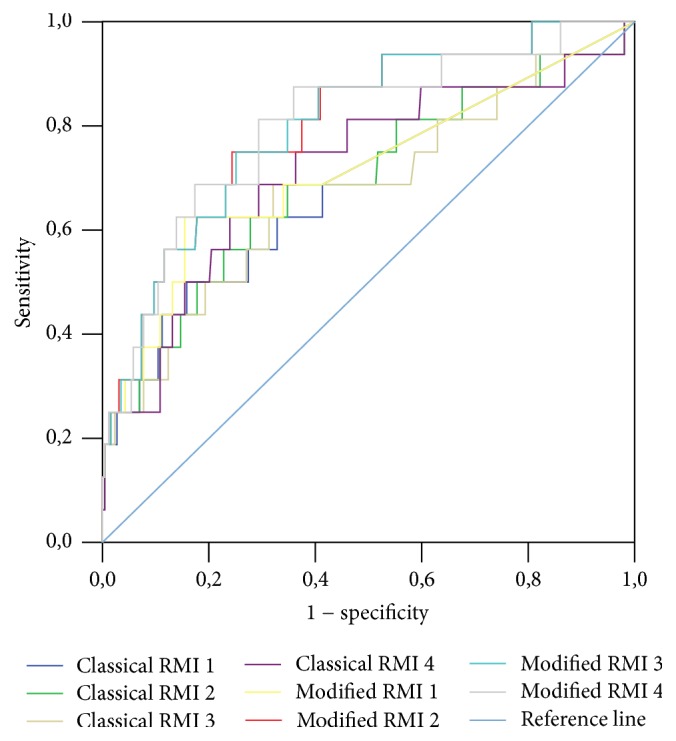
Diagnostic accuracy of classical and modified risk of malignancy indices (RMIs) 1–4 for differentiation of malignant stage I (FIGO) from nonmalignant adnexal masses. Modified RMIs were obtained by replacing cancer antigen 125 with human epididymis secretory protein 4 in the classical RMI. Diagnostic accuracy was based on receiver operating characteristic analysis. Diagonal segments are produced by ties.

A ROC curve was constructed for classical and mRMIs 1–4 obtained for the entire study sample, as well as for premenopausal and postmenopausal patients (Figures 1, 2, and 3, resp.). A ROC curve was also constructed for these diagnostic tools for differentiation of malignant stage I (FIGO) from nonmalignant adnexal masses (Figure 4). The DOR and AUC of the classical and modified indices for whole sample with corresponding *p* values are shown in Table 8. The DOR and AUC of diagnostic indices for differentiation of malignant stage I (FIGO) from nonmalignant adnexal tumors is shown in Table 9. The optimal cut-off levels of mRMI obtained by ROC-AUC were used for calculating DOR. Measures for diagnostic accuracy of mRMI for differentiation between all malignant and only stage I (FIGO) from nonmalignant adnexal tumors are displayed in Tables 10 and 11, respectively.

All classical and mRMIs were able to differentiate malignant from nonmalignant adnexal tumors. However, the AUCs for all mRMIs were higher when considering the entire study sample of 312 patients. Nonetheless, according to the Hanley and McNeil *p* values, the AUCs of corresponding classical and mRMIs 1–4 were not significantly different (Table 12) in the entire study sample or in either group defined in terms of menopausal status (i.e., premenopausal or postmenopausal patients) as well as in the differentiation of malignant stage I (FIGO) from nonmalignant adnexal masses. The optimal cut-off levels for mRMIs 1–4 for the whole population were 103.2, 250, 188, and 380, respectively. Lower and higher optimal cut-off levels were found in premenopausal and postmenopausal patients, respectively. Comparisons of predictive accuracy between classical and mRMIs 1–4 for differentiation of malignant stage I (FIGO) from nonmalignant adnexal tumors are presented in Table 12.

## 4. Discussion

With the development of 3D ultrasound, Doppler ultrasound, and novel tumor markers, it may be possible to further improve the accuracy of available RMIs. Wang et al. developed a binary logistic regression model to improve RMIs by incorporating tumor-specific growth factor and Doppler blood-flow parameters and found that, compared to RMI 1, the new RMI provided better predictions, especially in the diagnosis of ovarian germ cell tumors and other early-stage adnexal tumors [14].

In the present study, we considered cut-off values of 200 for RMIs 1–3 and 450 for RMI 4 according to the thresholds suggested by the original proponents of these indices. Some authors, however, have reported other values for optimal RMI thresholds when discriminating malignancy from nonmalignancy [15–19]. The discrepancies likely originate from differences in population composition, sample size, proportion of malignant pathologies, incidence of advanced malignant disease, proportion of menopausal patients, experience of the ultrasound examiner, type of medical center (oncologic or otherwise), and laboratory method for detecting CA125 [15–21]. It might be difficult to determine a cut-off value for RMIs with worldwide acceptance [22].

To our knowledge, this is the first study to analyze four variants of RMI in the Polish population. Our results revealed that all classical RMI variants could differentiate malignant from nonmalignant adnexal pathologies. Although our results agree with most previous studies in other populations, care should be taken when applying our findings in clinical practice involving other populations. For example, Ong et al. found that RMIs 1–4 could not appropriately distinguish between malignant and nonmalignant cases in an Asian population, although such findings may be attributed to the retrospective design of the study and the fact that endometriotic cysts accounted for the majority (76.8%) of nonmalignant lesions in the study population [23].

The RMI scoring systems combine several diagnostic parameters in order to improve the diagnostic performance of each individual parameter [24]. In our study, statistical analysis revealed that the incidence of malignancy increased significantly with increasing age, ultrasound score, and tumor diameter. Moreover, malignant disease was significantly more common among postmenopausal patients than among premenopausal patients. These findings are compatible with those of Yamamoto et al. [4]. Other mathematical models incorporating clinical data, ultrasound findings, and tumor markers have been proposed, such as the logistic regression model developed by the IOTA group (LR1, LR2) and the ADNEX model [25, 26]. However, LR− and ADNEX-based predictions refer to the probability of malignancy, whereas RMI-based predictions indicate that malignancy will occur if the RMI is above a certain cut-off point.

Ovarian cancer is one of the diagnostic dilemmas in gynecological oncology due to a lack of diagnostic tools for the early recognition of the disease [24]. If the diagnostic tool is highly sensitive, a higher proportion of malignant cases will be captured, which will eventually be managed appropriately (treatment by oncological gynecologists). The present study revealed relatively low sensitivities for diagnostic tools, which might be related to relatively high proportions of stage I (FIGO) adnexal malignancies (30%), where ultrasound features and tumor markers may not be differentiated from those of nonmalignant masses. However, high specificities emphasize the ability of the test to recognize subjects without the disease. The relatively low PPV and high NPV in the present study should be interpreted with caution and should not be transferred to other settings with different prevalence of the disease as the prevalence of the disease affects both of these values and PPV in particular [13]. Positive and negative LRs and DORs were useful in this study as they provided more estimates of the diagnostic value. DOR depends mainly on sensitivity and specificity but not on the prevalence of the disease [13]. The sensitivities of mRMIs were higher than those of the corresponding classical RMIs, especially in the premenopausal group. The higher sensitivity of mRMIs allows them to detect more malignant cases. However, lower specificity for mRMIs was found compared to the corresponding classical RMIs. Modifying RMIs resulted in a decreased positive likelihood ratio. Table 8 showed a comparison of the area under the curve and DOR. When looking at the whole sample, classical RMI and mRMI 4 had the highest AUC, while classical RMI 1 and mRMI 4 had the highest DOR. In premenopausal patients, classical RMI 1 and mRMI 3 had the highest AUC and DOR. In the postmenopausal group, classical RMI 1 had the highest AUC and DOR, mRMI 4 had the highest AUC, and mRMI 3 had the highest DOR.

For the differentiation of malignant stage I (FIGO) from nonmalignant adnexal tumors, mRMIs have higher sensitivities than classical RMIs, enabling proper detection of the early stages of malignancy. The AUC of mRMIs was higher than that of the classical RMIs but there was no significant difference between each mRMI and its corresponding classical RMI. In addition, the AUCs of classical RMIs and mRMIs for the differentiation of early stages of malignant adnexal tumors were lower than the values obtained for the whole population. Our results should be interpreted with caution, since there were a small number of patients with malignant stage I adnexal masses (*n* = 16).

Menopausal status is one of the key parameters in the RMI formula. The present study revealed that malignant pathologies occur more commonly in postmenopausal patients (*p* < 0.001). In the Polish population, 80% of ovarian cancer cases are noted in patients older than 50 years. However, ovarian cancer represents 4% of all malignant cases in patients aged ≤19 years and more than 6% in patients aged 20–44 years [27]. In the present study, the sensitivities of the four variants of classical and mRMIs were lower in premenopausal than in postmenopausal patients. The AUCs of the classical and modified variants of RMI were also lower in premenopausal patients. Overall, the AUCs of mRMIs and corresponding classical RMIs were not significantly different regardless of menopausal status.

Yenen et al. retrospectively compared 50 ovarian borderline tumors and 50 controls, reporting that RMI 4 was the best predictive RMI variant for the preoperative discrimination of such tumors, with a cut-off value of 200 [28]. It is difficult to interpret these results in the context of the present study because of the small sample size. Further prospective studies with higher numbers of patients are needed for more detailed evaluations.

The assessment of diagnostic performance using risk of ovarian malignancy algorithm (ROMA) and other predicting models was outside the scope of this study. One limitation of the present study was that the RMI-based predictions were not compared against those of other mathematical models or systemic scores. Anton et al. reported no differences in the accuracies of CA125, HE4, ROMA, and RMI for the differentiation of malignant from nonmalignant adnexal tumors [29]. Similarly, Karlsen et al. analyzed 1,218 patients with pelvic masses and concluded that ROMA and RMI perform equally well [30]. Another limitation of the present study is that it was conducted in a combined gynecology and oncology unit, where it is expected that patients with suspicion of malignant tumors are preferentially referred, potentially increasing the percentage of malignant adnexal pathologies in our study population. However, the proportion of malignant cases in our study population was low (16.67%), and 59.61% of the malignancies were diagnosed as stage III or IV, which are easily distinguished on ultrasound. Furthermore, we did not follow up the patients who were indicated for conservative management. Finally, the fact that we excluded patients with other diseases may have reduced the rate of false results. The present results should be verified by multicenter prospective studies to help in the selection of patients at the tertiary level for the most suitable surgical intervention. Our results are not representative of the whole population at the primary level, where the prevalence of the disease is low. However, this study was prospective in design, which represents its main strength, along with the fact that both tumor markers were measured at the same time using the same apparatus.

We found that replacing CA125 with HE4 did not improve the overall performance of RMIs. Previous studies showed that ultrasound parameters are superior to tumor markers [31, 32]. Taken together, these observations suggest that it might be more beneficial to modify RMI formulas to incorporate only ultrasound and clinical data without the use of tumor markers. Further prospective studies are needed to confirm this hypothesis and improve the diagnostic performance of RMIs.

## 5. Conclusions

Classical RMIs and mRMIs 1–4 are useful for the presurgical differentiation of malignant and nonmalignant adnexal tumors. Replacing CA125 with HE4 in the classical RMI formulas did not improve the diagnostic performance of these indices.

## Figures and Tables

**Table 1:** Distribution of nonmalignant adnexal pathologies.

Pathology	Number of patients (%)
Endometriotic cyst	58 (22.30%)
Dermoid cyst	54 (20.76%)
Simple cyst	52 (20%)
Serous cystadenoma	46 (17.69%)
Mucinous cystadenoma	19 (7.30%)
Tuboovarian abscess/hydrosalpinx	16 (6.15%)
Paraductal cyst	9 (3.46%)
Ovarian fibroma	6 (2.30%)

**Table 2:** Distribution of malignant adnexal masses.

Pathology	Number of patients (%)
Ovarian serous tumors	23 (44.23%)
Ovarian endometrioid tumors	11 (21.15%)
Ovarian borderline serous tumors	5 (9.62%)
Ovarian mucinous tumors	3 (5.77%)
Ovarian clear-cell tumors	3 (5.77%)
Oviduct malignancy	2 (3.85%)
Ovarian borderline mucinous tumors	2 (3.85%)
Ovarian sarcoma	1 (1.92%)
Ovarian folliculoma	1 (1.92%)
Ovarian germinal tumor	1 (1.92%)

**Table 3:** Distribution of FIGO stages of malignant adnexal pathologies.

FIGO stage	Number of patients (%)
IA	9 (17.30%)
IC	7 (13.46%)
IIA	4 (7.69%)
IIC	1 (1.92%)
IIIA	2 (3.85%)
IIIB	2 (3.85%)
IIIC	25 (48.08%)
IVB	2 (3.85%)

FIGO: Fédération Internationale de Gynécologie et d'Obstétrique.

**Table 4:** Distribution of age, menopause, ultrasound score, and maximum tumor diameter in patients with adnexal mass.

Parameter	Nonmalignant *n* = 260	Malignant *n* = 52	*p* value
Age, years			
<30	42 (16.2%)	1 (1.9%)	<0.001
31–40	68 (26.2%)	3 (5.8%)	
41–50	60 (23.1%)	7 (13.5%)	
>50	90 (34.6%)	41 (78.8%)	
Menopausal status			
Premenopausal	184 (70.8%)	11 (21.2%)	<0.001
Postmenopausal	76 (29.2%)	41 (78.8%)	
Ultrasound score			
≤1	227 (87.3%)	15 (28.8%)	<0.001
2–5	33 (12.7%)	37 (71.2%)	
Maximum tumor diameter			
<70 mm	169 (65.0%)	15 (28.8%)	<0.001
≥70 mm	91 (35.0%)	37 (71.2%)	

Data are shown as numbers and frequencies (percentage). *p* values were computed using the chi-square test.

**Table 5:** Descriptive statistics regarding the distribution of CA125 and HE4 levels in patients with adnexal mass.

Tumor marker	Parameter	Nonmalignant *n* = 260	Malignant *n* = 52
HE4, pmol/L	Mean	53.43	1133.56
	Median	46.55	373.40
	Minimum	17.80	38.30
	Maximum	192.30	8847.00

CA125, U/mL	Mean	41.22	743.63
	Median	20.50	282.40
	Minimum	4.34	6.32
	Maximum	506.80	9083.00

The serum levels of HE4 and CA125 were significantly higher among patients with malignant adnexal masses (*p* < 0.001). HE4, human epididymis secretory protein 4; CA125, cancer antigen 125

**Table 6:** Diagnostic performance of classical risk of malignancy indices (RMIs) 1–4 in patients with adnexal masses.

Group of patients	RMI variant	Diagnostic performance of RMI						
		Sensitivity (95% CI)	Specificity (95% CI)	PPV (95% CI)	NPV (95% CI)	LR+ (95% CI)	LR– (95% CI)	Diagnostic accuracy (95% CI)
Entire study sample	RMI 1	75.0% (63.2–86.7%)	95.3% (92.8–97.9%)	76.4% (64.8–88.1%)	95% (92.3–97.6%)	16.2 (9.1–28.8)	0.2 (0.1–0.4)	91.9% (88.9–95.0%)
	RMI 2	78.8% (67.7–89.9%)	87.6% (83.6–91.6%)	56.1% (44.7–67.5%)	95.3% (92.7–98.0%)	6.2 (4.3–8.9)	0.2 (0.1–0.4)	86.1% (82.3–90.0%)
	RMI 3	76.9% (65.4–88.3%)	91.8% (88.5–95.2%)	65.5% (53.6–77.4%)	95.2% (92.5–97.8%)	9.4 (6.1–14.6)	0.2 (0.1–0.4)	89.3% (85.9–92.8%)
	RMI 4	75% (63.2–86.7%)	92.2% (89–95.5%)	66.1% (54.0–78.1%)	94.8% (92.1–97.5%)	9.7 (6.1–15.2)	0.2 (0.1–0.4)	89.3% (85.9–92.8%)

Premenopausal patients	RMI 1	45.4% (16–74.8%)	96.7% (94.1–99.3%)	45.4% (16.0–74.8%)	96.7% (94.1–99.3%)	13.9 (5–38.6)	0.5 (0.3–0.9)	93.8% (90.4–97.2%)
	RMI 2	54.5% (25.1–83.9%)	92.3% (88.4–96.2%)	30% (9.9–50%)	97.1% (94.6–99.6%)	7.1 (3.4–14.9)	0.4 (0.2–0.9)	90.2% (86.0–94.3%)
	RMI 3	54.5% (25.1–83.9%)	92.8% (89.1–96.6%)	31.5% (10.6–52.4%)	97.1% (94.6–99.6%)	7.6 (3.6–16.2)	0.4 (0.2–0.9)	90.7% (86.6–94.8%)
	RMI 4	45.4% (16–74.8%)	94.5% (91.2–97.8%)	33.3% (9.4–57.1%)	96.6% (94.0–99.2%)	8.3 (3.4–20.1)	0.5 (0.3–0.9)	91.7% (87.8–95.6%)

Postmenopausal patients	RMI 1	82.9% (71.4–94.4%)	92.1% (86–98.1%)	85% (73.9–96.0%)	90.9% (84.4–97.3%)	10.5 (4.8–22.9)	0.1 (0.09–0.3)	88.8% (83.1–94.5%)
	RMI 2	85.3% (74.5–96.1%)	76.3% (66.7–85.8%)	66% (53.2–78.7%)	90.6% (83.4–97.7%)	3.6 (2.3–5.5)	0.1 (0.09–0.4)	79.4% (72.1–86.8%)
	RMI 3	82.9% (71.4–94.4%)	89.4% (82.5–96.3%)	80.9% (69.0–92.8%)	90.6% (84.0–97.2%)	7.8 (4–15.3)	0.1 (0.09–0.3)	87.1% (81.1–93.2%)
	RMI 4	82.9% (71.4–94.4%)	86.8% (79.2–94.4%)	77.2% (64.8–89.6%)	90.4% (83.6–97.1%)	6.3 (3.4–11.4)	0.19 (0.1–0.3)	85.4% (79.0–91.8%)

Data are shown with 95% confidence intervals (CIs). PPV: positive predictive value; NPV: negative predictive value; LR+: positive likelihood ratio; LR−: negative likelihood ratio.

**Table 7:** Diagnostic performance of classical RMIs 1–4 for differentiation of malignant stage I (FIGO) from nonmalignant adnexal tumors.

RMI variant	Diagnostic performance of RMI						
	Sensitivity (95% CI)	Specificity (95% CI)	PPV (95% CI)	NPV (95% CI)	LR+ (95% CI)	LR- (95% CI)	Diagnostic accuracy (95% CI)
RMI 1	25% (3.7–46.2%)	95.3% (92.8–97.9%)	25% (3.7–46.2%)	95.3% (92.8–97.9%)	5.4 (1.9–14.9)	0.7 (0.5–1)	91.3% (87.9–94.6%)
RMI 2	37.5% (13.7–61.2%)	87.6% (83.6–91.6%)	15.7% (4.1–27.3%)	95.7% (93.2–98.3%)	3 (1.4–6.1)	0.7 (0.4–1)	84.7% (80.4–88.9%)
RMI 3	31.2% (8.5–53.9%)	91.8% (88.5–95.2%)	19.2% (4–34.3%)	95.5% (93–98.1%)	3.8 (1.6–8.8)	0.7 (0.5–1)	88.3% (84.5–92.1%)
RMI 4	25% (3.7–46.2%)	92.2% (89–95.5%)	16.6% (1.7–31.5%)	95.2% (92.5–97.8)	3.2 (1.2–8.3)	0.8 (0.6–1)	88.3% (84.5–92.1%)

Data are shown with 95% confidence intervals (CIs). PPV: positive predictive value; NPV: negative predictive value; LR+: positive likelihood ratio; LR−: negative likelihood ratio; FIGO: Fédération Internationale de Gynécologie et d'Obstétrique.

**Table 8:** Diagnostic odds ratio (DOR) and area under the curve (AUC) for the predictive accuracy of classical and modified risk of malignancy indices (RMIs) 1–4 in patients with adnexal mass.

RMI variant	All patients				Premenopausal patients				Postmenopausal patients			
	DOR (95% CI)	AUC (95% CI)	*p* value	Optimal cut-off	DOR (95% CI)	AUC (95% CI)	*p* value	Optimal cut-off	DOR (95% CI)	AUC (95% CI)	*p* value	Optimal cut-off
RMI 1	62 (26.3–145.6)	0.899 (0.838–0.960)	<0.001	56	24.7 (5.8–104.2)	0.818 (0.654–0.982)	<0.001	19	56.6 (17.6–181.6)	0.906 (0.838–0.973)	<0.001	112
RMI 2	25.9 (12–55.7)	0.900 (0.840–0.960)	<0.001	123	14.4 (3.9–53.4)	0.798 (0.606–0.99)	0.001	59	18.7 (6.8–51.8)	0.895 (0.822–0.969)	<0.001	245
RMI 3	37.7 (17.2–82.7)	0.895 (0.832–0.957)	<0.001	107	15.6 (4.2–58.3)	0.795 (0.604–0.986)	0.001	58	41.2 (13.8–123.3)	0.896 (0.823–0.970)	<0.001	168
RMI 4	35.8 (16.5–77.8)	0.908 (0.851–0.966)	<0.001	219	14.4 (3.7–55.4)	0.802 (0.604–1.000)	0.001	110	32 (11.2–91.6)	0.906 (0.842–0.971)	<0.001	315
mRMI 1	31.1 (13.6–71.2)	0.903 (0.844–0.963)	<0.001	103.2	6.9 (1.7–27.2)	0.839 (0.676–1.000)	<0.001	40	31.1 (10.7–90)	0.907 (0.841–0.973)	<0.001	330
mRMI 2	30.2 (13.2–69)	0.929 (0.886–0.972)	<0.001	250	20.5 (4.2–99.7)	0.875 (0.747–1.000)	<0.001	69	31.1 (10.7–90)	0.923 (0.869–0.978)	<0.001	770
mRMI 3	30.2 (13.2–69)	0.930 (0.887–0.973)	<0.001	188	20.5 (4.2–99.7)	0.876 (0.748–1.000)	<0.001	69	34.4 (11.7–101.1)	0.924 (0.870–0.979)	<0.001	480
mRMI 4	41.3 (17.2–98.8)	0.931 (0.887–0.976)	<0.001	380	19.8 (4.1–96.0)	0.856 (0.707–1.000)	<0.001	118	23.5 (8.5–64.5)	0.930 (0.883–0.977)	<0.001	1300

Modified RMIs (mRMIs) were obtained by replacing cancer antigen 125 with human epididymis secretory protein 4 in the classical RMI. Data are shown with 95% confidence intervals (CIs).

**Table 9:** Diagnostic odds ratio (DOR) and area under the curve (AUC) for the predictive accuracy of classical and modified risk of malignancy indices (RMIs) 1–4 for the differentiation of malignant stage I (FIGO) from nonmalignant adnexal tumors.

RMI variant	DOR (95% CI)	AUC (95% CI)	*p* value	Optimal cut-off
RMI 1	6.8 (1.9–24.5)	0.686 (0.535–0.837)	0.013	12.5
RMI 2	4.2 (1.4–12.5)	0.692 (0.541–0.843)	0.010	58
RMI 3	5.1 (1.6–16.2)	0.674 (0.519–0.830)	0.019	53
RMI 4	3.9 (1.1–13.4)	0.717 (0.570–0.864)	0.004	83
mRMI 1	4.2 (1.4–12.5)	0.715 (0.561–0.869)	0.004	38
mRMI 2	9.1 (2.8–29.4)	0.802 (0.690–0.913)	<0.001	177
mRMI 3	6.6 (2.2–19.7)	0.803 (0.692–0.914)	<0.001	135
mRMI 4	7.2 (2.2–23.2)	0.801 (0.682–0.920)	<0.001	205

Modified RMIs (mRMIs) were obtained by replacing cancer antigen 125 with human epididymis secretory protein 4 in the classical RMI. Data are shown with 95% confidence intervals (CIs). FIGO: Fédération Internationale de Gynécologie et d'Obstétrique.

**Table 10:** Diagnostic performance of modified risk of malignancy indices (mRMI) 1–4 in patients with adnexal masses.

Group of patients	mRMI variant	Diagnostic performance of RMI						
		Sensitivity (95% CI)	Specificity (95% CI)	PPV (95% CI)	NPV (95% CI)	LR+ (95% CI)	LR− (95% CI)	Diagnostic accuracy (95% CI)
Entire study sample	mRMI 1	84.6% (74.8–94.4%)	85% (80.6–89.3%)	53% (42.2–63.7%)	96.5% (94.1–98.8%)	5.6 (4.1–7.7)	0.1 (0.09–0.3)	84.9% (80.9–88.9%)
	mRMI 2	84.6% (74.8–94.4%)	84.6% (80.2–89%)	52.3% (41.7–63%)	96.4% (94.1–98.8%)	5.5 (4–7.4)	0.1 (0.09–0.3)	84.6% (80.6–88.6%)
	mRMI 3	84.6% (74.8–94.4%)	84.6% (80.2–89%)	52.3% (41.7–63%)	96.4% (94.1–98.8%)	5.5 (4–7.4)	0.1 (0.09–0.3)	84.6% (80.6–88.6%)
	mRMI 4	86.5% (77.2–95.8%)	86.5% (82.3–90.6%)	56.2% (45.3–67.1%)	96.9% (94.7–99.1%)	6.4 (4.6–8.9)	0.1 (0.07–0.3)	86.5% (82.7–90.3%)

Premenopausal patients	mRMI 1	72.7% (46.4–99%)	72.2% (65.8–78.7%)	13.5% (4.8–22.2%)	97.7% (95.3–100%)	2.6 (1.7–4)	0.3 (0.1–0,9)	72.3% (66–78.5%)
	mRMI 2	81.8% (59–100%)	82% (76.5–87.6%)	21.4% (9–33.8%)	98.6% (96.8–100%)	4.5 (3–6.9)	0.2 (0.06–0.7)	82% (76.6–87.4%)
	mRMI 3	81.8% (59–100%)	82% (76.5–87.6%)	21.4% (9–33.8%)	98.6% (96.8–100%)	4.5 (3–6.9)	0.2 (0.06–0.7)	82% (76.6–87.4%)
	mRMI 4	81.8% (59–100%)	81.5% (75.9–87.1%)	20.9% (8.7–33%)	98.6% (96.8–100%)	4.4 (2.9–6.6)	0.2 (0.06–0.7)	81.5% (76–86.9%)

Postmenopausal patients	mRMI 1	85.3% (74.5–96.1%)	84.2% (76–92.4%)	74.4% (62–86.9%)	91.4% (84.8–97.9%)	5.4 (3.1–9.2)	0.1 (0.08–0.3)	84.6% (78–91.1%)
	mRMI 2	85.3% (74.5–96.1%)	84.2% (76–92.4%)	74.4% (62–86.9%)	91.4% (84.8–97.9%)	5.4 (3.1–9.2)	0.1 (0.08–0.3)	84.6% (78–91.1%)
	mRMI 3	85.3% (74.5–96.1%)	85.5% (77.6–93.4%)	76% (63.7–88.4%)	91.5% (85–98%)	5.8 (3.3–10.3)	0.1 (0.08–0.3)	85.4% (79–91.8%)
	mRMI 4	82.9% (71.4–94.4%)	82.8% (74.4–91.3%)	72.3% (59.5–85.1%)	90% (82.9–97%)	4.8 (2.8–8.1)	0.2 (0.1–0.4)	82.9% (76–89.7%)

Modified RMIs (mRMIs) were obtained by replacing CA125 with HE4 in the classical RMI. Data are shown with 95% confidence intervals (CIs). PPV: positive predictive value; NPV: negative predictive value; LR+: positive likelihood ratio; LR−: negative likelihood ratio.

**Table 11:** Diagnostic performance of modified risk of malignancy indices (mRMIs) 1–4 for the differentiation of malignant stage I (FIGO) from nonmalignant adnexal tumors.

RMI variant	Diagnostic performance of mRMI						
	Sensitivity (95% CI)	Specificity (95% CI)	PPV (95% CI)	NPV (95% CI)	LR+ (95% CI)	LR− (95% CI)	Diagnostic accuracy (95% CI)
mRMI 1	68.7% (46–91.4%)	65.7% (60–71.5%)	11% (4.8–17.1%)	97.1% (94.7–99.6%)	2 (1.3–2.9)	0.4 (0.2–0.9)	65.9% (60.3–71.5%)
mRMI 2	75% (53.7–96.2%)	75.3% (70.1–80.6%)	15.7% (7.5–23.9%)	98% (96–99.9%)	3 (2.1–4.3)	0.3 (0.1–0.7)	75.3% (70.2–80.4%)
mRMI 3	68.7% (46–91.4%)	75% (69.7–80.2%)	14.4% (6.5–22.3%)	97.5% (95.3–99.6%)	2.7 (1.8–4)	0.4 (0.2–0.8)	74.6% (69.5–79.7%)
mRMI 4	75% (53.7–96.2%)	70.7% (65.2–76.2%)	13.6% (6.4–20.8%)	97.8% (95.8–99.9%)	2.5 (1.8–3.6)	0.3 (0.1–0.8)	71% (65.6–76.3%)

Modified RMIs (mRMIs) were obtained by replacing cancer antigen 125 with human epididymis secretory protein 4 in the classical RMI. Data are shown with 95% confidence intervals (CIs). PPV: positive predictive value; NPV: negative predictive value; LR+: positive likelihood ratio; LR−: negative likelihood ratio; FIGO: Fédération Internationale de Gynécologie et d'Obstétrique.

**Table 12:** Comparison of predictive accuracy between classical and modified risk of malignancy indices (RMIs) 1–4 in patients with adnexal masses.

Compared RMI variants	*p* value			
	Differentiation of malignant (*n* = 52) from nonmalignant (*n* = 260) adnexal tumors			Differentiation of malignant stage I (FIGO) (*n* = 16) from nonmalignant (*n* = 260) adnexal tumors
	All patients (*n* = 312)	Premenopausal (*n* = 117)	Postmenopausal (*n* = 195)	
mRMI 1 versus RMI 1	*p* = 0.923	*p* = 0.848	*p* = 0.983	*p* = 0.784
mRMI 2 versus RMI 2	*p* = 0.452	*p* = 0.471	*p* = 0.543	*p* = 0.276
mRMI 3 versus RMI 3	*p* = 0.368	*p* = 0.449	*p* = 0.541	*p* = 0.203
mRMI 4 versus RMI 4	*p* = 0.540	*p* = 0.620	*p* = 0.585	*p* = 0.402

Modified RMIs (mRMIs) were obtained by replacing cancer antigen 125 with human epididymis secretory protein 4 in the classical RMIs. Predictive accuracy was expressed in terms of the area under the receiver operating characteristic curve. We obtained *p* values using the Hanley and McNeil method.
